# Supplementary material for: Predictors and Long-Term Prognostic Significance of Acute Renal Function Change in Patients Who Underwent Surgical Aortic Valve Replacement
Source: J Clin Med. 2023 Jul 27;12(15):4952. doi: 10.3390/jcm12154952 (PMC10419392; doi:10.3390/jcm12154952)
Supplement: Supplementary file 1 [file jcm-12-04952-s001.zip › jcm-2491328-supplementary.pdf]

Table S1. Binary logistic regression results for renal improvement and renal deterioration by 10% change in eGFR.

Predictors of renal improvement	Adjusted odds ratios (95% CI) Odds ratio (95% CI)	P value
**Preoperative stage 1 CKD[a]**	**0.070 (0.041, 0.117)**	**<0.001**
**Preoperative stage 3 CKD[a]**	**3.402 (2.279, 5.078)**	**<0.001**
**Preoperative stage 4 CKD[a]**	**5.486 (2.046, 14.711)**	**<0.001**
Biological vs mechanical valve	0.951 (0.682, 1.326)	0.768
Diabetes	0.750 (0.511, 1.101)	0.142
Previous malignancy	1.059 (0.558, 2.012)	0.860
Hyperlipidemia	1.108 (0.639, 1.919)	0.716
Current smoker	1.423 (0.994, 2.037)	0.054
Atrial fibrillation	0.992 (0.693, 1.422)	0.967
Hypertension	0.678 (0.448, 1.027)	0.066
Cerebral vascular accident	1.466 (0.920, 2.336)	0.108
Peripheral vascular disease	0.654 (0.366, 1.166)	0.150
Chronic obstructive pulmonary disease	0.959 (0.567, 1.623)	0.877
Liver disease	2.148 (0.846, 5.450)	0.108
Female	1.137 (0.782, 1.652)	0.502
**Age (years)**	**0.952 (0.936, 0.967)**	**<0.001**
Preoperative glucose (mg/dl)	1.004 (0.999, 1.008)	0.102
Preoperative hemoglobin (g/dl)	1.035 (0.920, 1.165)	0.564
Preoperative platelets (10^3/ µl)	1.003 (1.000, 1.005)	0.053
**Predictors of renal deterioration**	**Adjusted odds ratios (95% CI) Odds ratio (95% CI)**	**P value**
**Preoperative stage 2 CKD[b]**	**1.992 (1.611, 2.463)**	**<0.001**
**Preoperative stage 3 CKD[b]**	**2.673 (1.961, 3.642)**	**<0.001**
**Preoperative stage 4 CKD[b]**	**2.862 (1.220, 6.712)**	**0.016**
**Biological vs mechanical valve**	**0.616 (0.509, 0.744)**	**<0.001**
Diabetes	0.976 (0.788, 1.207)	0.820
Previous malignancy	0.877 (0.606, 1.268)	0.485
Hyperlipidemia	1.158 (0.839, 1.597)	0.372
Current smoker	0.983 (0.802, 1.204)	0.866
**Atrial fibrillation**	**1.249 (1.027, 1.520)**	**0.026**
Hypertension	0.983 (0.779, 1.239)	0.883
Cerebral vascular accident	1.045 (0.796, 1.372)	0.753
Peripheral vascular disease	1.086 (0.802, 1.472)	0.592
Chronic obstructive pulmonary disease	0.980 (0.729, 1.318)	0.894
Liver disease	0.776 (0.402, 1.498)	0.449
Female	1.087 (0.888, 1.331)	0.419
**Age (years)**	**1.024 (1.014, 1.034)**	**<0.001**
Preoperative glucose (mg/dl)	1.000 (0.997, 1.002)	0.886
**Preoperative hemoglobin (g/dl)**	**0.831 (0.772, 0.895)**	**<0.001**
**Preoperative platelets (10^3/ µl)**	**0.998 (0.996, 0.999)**	**0.003**

Abbreviations: CKD (chronic kidney disease), PVD (peripheral vascular disease), eGFR (estimated glomerular filtration rate).

Table S2. Change in post-operative CKD stage by pre-operative CKD stage.

Preoperative CKD stage	Deteriorated by 3 stages	Deteriorated by 2 stages	Deteriorated by 1 stage	Unchanged stage	Improved by 1 stage	Improved by 2 stages
**Stage 4**	0	0	9	22	9	1
**Stage 3**	0	8	65	220	57	2
**Stage 2**	5	24	334	598	83	0
**Stage 1**	1	34	333	597	0	0
**Total**	6	66	741	1437	149	3

Table S3. Mortality rates one week post op.

Preoperative CKD stage	One week post op mortality n/total (%)
**Stage 1**	9/974 (0.92%)
**Stage 2**	5/1049 (0.48%)
**Stage 3**	6/358 (1.68%)
**Stage 4**	1/42 (2.38%)

Chi squared comparison of one week mortality p=0.132.

Table S4. Paired sample T-test comparing pre- and post- operative eGFR change by preoperative CKD stage.

Preoperative CKD stage	Mean eGFR change	P-value
**Stage 1**	-8.21 (SD 12.71)	<0.001
**Stage 2**	-11.31 (SD 16.86)	<0.001
**Stage 3**	-5.36 (SD 15.92)	<0.001
**Stage 4**	-1.42 (SD 10.58)	0.394

Table S5. Tukey post hoc testing comparing mean eGFR change between the groups, ANOVA p<0.001.

(I) CKD STAGE	(II) CKD STAGE	MEAN DIFFERENCE (I-II)	P VALUE
**STAGE 4**	Stage 3	3.93975	.389
	Stage 2	9.88945	<.001
	Stage 1	6.78576	.025
**STAGE 3**	Stage 4	-3.93975	.389
	Stage 2	5.94970	<.001
	Stage 1	2.84601	.013
**STAG 2**	Stage 4	-9.88945	<.001
	Stage 3	-5.94970	<.001
	Stage 1	-3.10369	<.001
**STAGE 1**	Stage 4	-6.78576	.025
	Stage 3	-2.84601	.013
	Stage 2	3.10369	<.001

Supplemental Materials

Covariates included in regression analyses:

Age, sex, pre-operative CKD stage, mechanical/biological valve, body mass index as well as comorbidities including diabetes, previous malignancy, hyperlipidemia, current smokers, atrial fibrillation, hypertension, cerebral vascular accident, peripheral vascular disease, chronic obstructive pulmonary disorder, and liver disease. Pre-operative serum lab values were also included, including glucose, hemoglobin, and platelets.

Covariates included in the Cox hazard models:

Age, sex, change in renal function, pre-operative CKD stage, biological/mechanical valve as well as comorbidities including diabetes, previous malignancy, hyperlipidemia, current smokers, atrial fibrillation, hypertension, cerebral vascular accident, peripheral vascular disease, chronic obstructive pulmonary disorder, and liver disease.

Supplemental analysis of outcome by 10% or greater change in eGFR

Higher baseline CKD stages were more likely to show a 10% improvement in eGFR: 29.3% of stage 4, 21.9% of stage 3, 11.2% of stage 2 and 3% of stage 1. While lower baseline CKD stages were more likely to show a post-SAVR deterioration in CKD stage, baseline stages 2, 3 and 4 were more likely to suffer from a 10% decrease in eGFR than baseline stage 1 patients: 53.1%, 52.6% and 51.2% compared to 34.7%, respectively.

Predictors of improvement and deterioration in renal function by 10% change in eGFR

Variables associated with a 10% increase in eGFR also included smoking ($p=0.039$), liver disease ($p=0.018$), higher baseline CKD stage ($p<0.001$), lower age ($p<0.001$), lack of diabetes ($p=0.02$) and higher baseline hemoglobin ($p=0.042$), but also included higher baseline platelet and glucose levels ($p=0.008$ and $p=0.023$, respectively).

Deterioration in CKD stage was associated with a biological valve ($p=0.001$), atrial fibrillation ($p<0.001$), peripheral vascular disease ($p=0.029$), lower baseline hemoglobin ($p<0.001$), baseline stage 1 CKD ($p=0.002$ compared to baseline stage 4) and higher age ($p<0.001$). Variables associated with a 10% decrease in eGFR were implantation of a biological valve ($p<0.001$), atrial fibrillation ($p=0.002$), low baseline hemoglobin ($p<0.001$), baseline stage 1 CKD ($p=0.002$ compared to baseline stage 4), lower baseline platelets ($p=0.002$) and older age ($p<0.001$).
